# Socioeconomic Disparities in the Demand for and Use of Virtual Visits Among Senior Adults During the COVID-19 Pandemic: Cross-sectional Study

**DOI:** 10.2196/35221

**Published:** 2022-03-22

**Authors:** Ellie Yu, Simon Hagens

**Affiliations:** 1 Canada Health Infoway Toronto, ON Canada

**Keywords:** virtual care, virtual visit, COVID-19, survey, virtual care demand, virtual care use, older adults, elderly care, aging, digital health, pandemic

## Abstract

**Background:**

The COVID-19 pandemic has limited the provision of in-person care and accelerated the need for virtual care. Older adults (65+ years) were 1 of the highest user groups of in-person health care services prior to the pandemic. Social distancing guidelines and high rates of mortality from coronavirus infections among older adults made receiving in-person health care services challenging for older adults. The provision of virtual care technologies can help to ensure continuity of care and provide essential health care services during the pandemic to those at high risk of contracting the COVID-19 coronavirus, including older adults. It is also essential to understand and address potential socioeconomic, demographic, and health disparities in the demand for and use of virtual care technologies among older adults.

**Objective:**

The objective of this study is to investigate socioeconomic disparities in the demand for and use of virtual visits during the COVID-19 pandemic among older adults in Canada.

**Methods:**

A cross-sectional web survey was conducted with 12,052 Canadians over the age of 16 years, selected from Leger’s Léger Opinion panel from July 14 to August 6, 2021. Associations between socioeconomic factors and the demand for and use of virtual visits were tested using χ2 tests and logistic regression models for telephone visits, video visits, and secure messaging. Weighting was applied using the 2016 census reference variables to render a representative sample of the Canadian population.

**Results:**

A total of 2303 older adults were surveyed. Older adults expressed the highest demand for and use of telephone visits, following by video visits and secure messaging. eHealth literacy was positively associated with the use of all 3 virtual care modalities. Higher income was negatively associated with the use of video visits (odds ratio [OR] 0.65, 95% CI 0.428-0.974, *P*=.03). Having no private insurance coverage was negatively associated with use of secure messaging (OR 0.73, 95% CI 0.539-0.983, *P*=.04), but living in a rural community (OR 0.172, 95% CI 1.12-2.645, *P*=.01) and being born outside of Canada (OR 0.150, 95% CI 1.041-2.173, *P*=.03) were positively associated with the use of secure messaging. Higher education (OR 0.078, 95% CI 0.633-0.97, *P*=.02) and being non-White (OR=0.054, 95% CI 0.312-0.92, *P*=.02) were negatively associated with the use of the telephone.

**Conclusions:**

This study found that compared to video visits and secure messaging, the demand for and use of telephone visits were more prevalent among older adults during the pandemic. The gaps between the demand for and use of video and secure messaging services remain substantial. Our results highlight socioeconomic disparities among older adults that could potentially explain this trend. Lower income and a lower education level may act as barriers for older adults in acquiring the skills and technologies necessary to use more complex solutions, such as video and secure messaging. In addition, higher eHealth literacy was found to be critical for older adults to successfully navigate all types of virtual visit technologies.

## Introduction

The COVID-19 pandemic has posed unprecedented challenges for Canadians and the Canadian health care system. As in-person visits halted due to social distancing guidelines in the beginning of 2020, there has been a rapid expansion and uptake of virtual care technologies by the health care system to meet patient needs. Virtual care technologies can be defined as “any interaction between patients and/or members of their circle of care, occurring remotely, using any forms of communication or information technologies, with the aim of facilitating or maximizing the quality and effectiveness of patient care” [1]. Virtual care can be conducted via virtual visits through modalities, including telephone visits, video visits, or secure messaging interactions with a health care provider. Virtual visits have the potential to expand access to care for patients, especially during the COVID-19 pandemic. In Ontario, primary care provision quickly shifted to virtual with a 56-fold increase in virtual visits between March and July 2020 [2]. Between January and August 2021, virtual visits accounted for 35% of all most recent patient-reported visits, with 78% conducted via the telephone, 19% via videoconferencing, and 3% via secure messaging [3].

The declaration of the COVID-19 pandemic and social distancing measures disproportionally affected older adults above the age of 65 years. Older adults were particularly affected by COVID-19 due to prevalent health conditions related to age that lead to more severe clinical outcomes when infected with COVID-19. Data from Statistics Canada indicate that between March 2020 and May 2021, older adults accounted for 93% of the deaths attributed to COVID-19 [4]. Prior to the pandemic, older adults were among the highest health service users [5,6]. Age-related health factors, such as multimorbidity and limitations on functional capacity, make routine as well as urgent health care critical for the well-being of older adults. Older adults may experience challenges with accessing health services due to physical capacity limitations, financial barriers, and deteriorating psychological conditions [7]. The COVID-19 pandemic posed additional challenges for older adults, such as misinformation, physical and psychological isolation, and limitations to routine activities [8].

There is a paucity of research that assessed the impact of COVID-19 on health system utilization by older adults and the barriers and facilitators related to the demand for and use of virtual visits. International studies have indicated that during the COVID-19 pandemic, the number of routine health care services have decreased for older adults [9]. Findings from the recent Commonwealth Fund survey on older adults suggest as many as 32% of Canadian older adults with multiple chronic conditions had to cancel or postpone at least 1 appointment due to the pandemic [10]. Evidence emerging from Ontario suggests that because of a quick shift to virtual care, older adults maintained higher levels of care during the pandemic despite the absence of in-person care in many care settings [2]. Presently, there is a lack of knowledge about how older adults have navigated the health care system during the pandemic and their demand for and use of virtual visits to substitute or supplement in-person care. The purpose of this study is to investigate potential disparities in the demand for and use of virtual visits among older adults by assessing the associations between socioeconomic characteristics of older adults with self-reported demand for and use of telephone visits, video visits, and secure messaging services during the COVID-19 pandemic.

## Methods

### Study Population and Design

The 2021 Canadian Digital Health Survey is a cross-sectional, web-based survey of 12,052 Canadians, conducted in both English and French through computer-assisted web interviewing technology. The survey was commissioned by Canada Health Infoway (Infoway) and conducted by Léger. The Léger Opinion (LEO) panel was used for this survey. LEO is Léger’s proprietary panel with nearly 500,000 representative panelists from all regions of Canada based on a representative sample of Canadian citizens with internet access. LEO's panelists were randomly selected using random digital dial samples, and panelists from more hard-to-reach target groups were also added to the panel through targeted recruitment campaigns. The survey questionnaire was created by Infoway, but the administration of the survey and the cleaning and coding of the survey data were conducted by Léger and then transferred to Infoway for analyses. A consent statement was presented to respondents at the beginning of the survey, and informed consent was obtained as part of the survey. No personal identifier was included in the data set to Infoway, and no personal health information was collected as part of the survey. Testing of the online survey was conducted by both Infoway as well as Léger staff. A small monetary incentive was offered to survey participates by Léger. The survey collected questions on demographic, socioeconomic and health characteristics as well as the self-reported demand for, and use of, digital health services, among other questions. In total, 68 questions were included in the survey.

Based on the respondent’s default language of choice, the survey was presented to respondents in either English or French. Data collection took place from July 14 to August 6, 2021. Using 2016 Canadian Census data, Leger’s methodologists applied weighting according to region, age, and gender to render a representative sample of the Canadian population. A margin of error cannot be associated with a nonprobability sample in a panel survey. For comparison purposes, a probability sample of this size would have a margin of error of ±0.895%, 19 times out of 20. For more details on the survey, please refer to a comprehensive report published by Infoway [3].

### Measures

Use of virtual visits was determined by the self-reported answer to the question “Have you used _____ in the last 12 months?” and the demand for virtual care was determined by the self-reported answer to the question “Are you interested in accessing _____, whether you currently have access or not?” The demand for and use of telephone visits, video visits, and secure messaging were assessed separately with the same questions.

#### Socioeconomic Factors

Tables 1-4 outline the socioeconomic, health-related, and demographic factors, as well as the eHealth Literacy Scale (eHEALS) used to measure eHealth literacy, respectively. Household income reports the self-reported household income before tax in the past year. Education level reports the self-reported highest level of education obtained, including qualification obtained outside of Canada, and responses were collapsed into secondary education or less; college diploma or trade certificates; undergraduate degree; graduate degrees, including paramedical professional degrees; other; none of the above; and prefer not to answer. Community size was measured using the question “How would you describe the community you live in?” and responses were rural, small, medium, or large population centers, and urban centers. The population size for a rural community was defined as less than 1000 people. The population size for small, medium, and large population centers was defined as 1000-19,999 people, 20,000-99,999 people, and 100,000-999,999 people, respectively. An urban center was defined as 1 million people and over. Immigrant status was assessed with the question “Are you a Canadian citizen?” Language at home reports the language spoken on a regular basis at home. Employment status reports the current employment status, and responses were collapsed into working, including full- and part-time employment; unemployed, including homemakers, disabled, and students; retired; other; and prefer not to answer. Health coverage was based on the question “Which of the following best describes the type of health insurance coverage you currently have?” and the categories were collapsed into public coverage only, private coverage, no coverage, and don’t know and prefer not to say. Private coverage includes insurance plans paid for by the respondent, a family member, an employer, or an association.

**Table 1:** Socioeconomic characteristics of older adults and bivariate associations with the demand for and use of virtual visits, 2011 Canadian Digital Health Survey (N=2303).

Factors		All n (unweighted, weighted)=(2303, 2454), weighted %^a^	Use^b^ n (unweighted, weighted)=(1149, 1245), weighted %	Versus have not used, *χ*^2^ (*df*)	*P* value	Demand^c^ n (unweighted, weighted)=(1773, 1902), weighted %	Versus have no interest, *χ*^2^ (*df*)	*P* value
**Household income (CAD $)^d^**		N/A^e^	N/A	12.5 (7)	.08	N/A	29.9 (7)	<.001
	24,999 or less	9.7	8.8	N/A	N/A	8.5	N/A	N/A
	25,000-49,999	22.9	21.8	N/A	N/A	21.9	N/A	N/A
	50,000-79,999	23	22.3	N/A	N/A	22.9	N/A	N/A
	80,000-99,999	14.1	15.6	N/A	N/A	14.9	N/A	N/A
	100,000-149,999	12.7	12.5	N/A	N/A	13.3	N/A	N/A
	150,000-249,999	4.8	5.5	N/A	N/A	5.4	N/A	N/A
	250,000 or more	1.0	1.2	N/A	N/A	1.1	N/A	N/A
	Prefer not to answer	11.9	12.3	N/A	N/A	12	N/A	N/A
**Education level**		N/A	N/A	21.2 (6)	.01	N/A	39.9 (6)	<.001
	Secondary or less	22.3	20.9	N/A	N/A	20.8	N/A	N/A
	College or trade	32.1	31.3	N/A	N/A	31.5	N/A	N/A
	Undergraduate degree	29.3	30.6	N/A	N/A	30.7	N/A	N/A
	Graduate degree or more	13.4	15.1	N/A	N/A	14.7	N/A	N/A
	Other	1.1	1.0	N/A	N/A	1.2	N/A	N/A
	None of the above	1.3	1.0	N/A	N/A	1	N/A	N/A
	Prefer not to answer	0.4	0.1	N/A	N/A	0.2	N/A	N/A
**Community size**		N/A	N/A	10.5 (4)	.03	N/A	0.6 (4)	.97
	Rural	10	9.9	N/A	N/A	10	N/A	N/A
	Small population center	18.2	16.6	N/A	N/A	18	N/A	N/A
	Medium population center	19.6	18.4	N/A	N/A	19.5	N/A	N/A
	Large population center	29.9	30.9	N/A	N/A	30.1	N/A	N/A
	Urban center	22.3	24.2	N/A	N/A	22.4	N/A	N/A
**Immigration status**		N/A	N/A	1.4 (2)	.50	N/A	2.7 (2)	.26
	Canadian citizen by birth	82.9	82.3	N/A	N/A	82.6	N/A	N/A
	Canadian citizen by naturalization^f^	16.0	16.8	N/A	N/A	16.5	N/A	N/A
	Not a Canadian citizen	1.0	0.9	N/A	N/A	0.9	N/A	N/A
**Language spoken at home**		N/A	N/A	12. 6 (2)	.01	N/A	1.5 (2)	.68
	English	76.1	78.4	N/A	N/A	76.6	N/A	N/A
	French	22.0	98.6	N/A	N/A	21.6	N/A	N/A
	Other	1.9	1.4	N/A	N/A	1.8	N/A	N/A
**Employment status**		N/A	N/A	7.9 (4)	.44	N/A	2.2 (4)	.97
	Working full-time or part-time	13.5	13.1	N/A	N/A	13.7	N/A	N/A
	Unemployed	2.0	2.0	N/A	N/A	2.1	N/A	N/A
	Retired	83.2	83.6	N/A	N/A	83.1	N/A	N/A
	Other	1.1	1.4	N/A	N/A	1.1	N/A	N/A
	Prefer not to answer	0	0	N/A	N/A	0	N/A	N/A
**Health care coverage**		N/A	N/A	74.3 (4)	<.001	N/A	28.2 (4)	<.001
	Public coverage only	54.5	61.1	N/A	N/A	56.2	N/A	N/A
	Private coverage	36.6	34.0	N/A	N/A	36.2	N/A	N/A
	No coverage	6.4	3.8	N/A	N/A	5.4	N/A	N/A
	I don't know	1.7	0.6	N/A	N/A	1.3	N/A	N/A
	Prefer not to answer	0.9	0.5	N/A	N/A	0.8	N/A	N/A

^a^Percentages are weighted and have been rounded and may not total 100.

^b^Older adults who have used of at least 1 type of virtual visit modality in the past 12 months.

^c^Older adults who have expressed the demand for at least 1 type of virtual visit modality.

^d^A currency exchange rate of CAD $1=US $0.78 is applicable.

^e^N/A: not applicable.

^f^Data not available due to low cell count in response categories.

**Table 2:** Health-related characteristics of older adults and bivariate associations with the demand for and use of virtual visits, 2011 Canadian Digital Health Survey (N=2303).

Factors			All n (unweighted, weighted)=(2303, 2454), weighted %^a^		Use^b^ n (unweighted, weighted)=(1149, 1245), weighted %		Versus have not used, *χ*^2^ (*df*)		*P* value		Demand^c^ n (unweighted, weighted)=(1773, 1902), weighted %		Versus have no interest, *χ*^2^ (*df*)		*P* value
**Access to a family physician**			N/A^d^		N/A		28.2 (1)		<.001		N/A		32.8 (1)		<.001
	Yes	93.9		96.3		N/A				95.0		N/A			
**Chronic conditions**															
	Chronic pain	15.5		18.3		14.5 (1)		<.001		16.7		8.4 (1)		.004	
	Cancer	5.4		5.5		0.1 (1)		.79		5.6		0.7 (1)		.40	
	Diabetes of all types	17.1		19.7		12.9 (1)		<.001		16.9		0.2 (1)		.63	
	Chronic obstructive pulmonary disease (COPD)	4.9		5.3		1.11 (1)		.30		5.0		0.4 (1)		.53	
	Arthritis	30.1		34.6		24.8 (1)		.001		30.9		2.9 (1)		.09	
	Cardiovascular disease (CVD)	7.3		8.4		5.2 (1)		.02		7.5		0.6 (1)		.45	
	Alzheimer disease or any other dementia	0.2		0.3		N/A		N/A		0.3		N/A		N/A	
	Developmental disability	0		0.1		N/A		N/A		0		N/A		N/A	
	Drug or alcohol dependency	1.0		1.5		4.6 (1)		.04		1.3		5.2 (1)		.02	
	Obesity	11.5		13.7		12.0 (1)		.001		12.3		4.9 (1)		.03	
	Learning disability	0.6		0.6		0.1 (1)		.80		0.7		N/A		N/A	
	Emotional, psychological, or mental health conditions	7.2		8.9		11.5 (1)		.001		7.9		5.5 (1)		.02	
	Physical disability	7.1		8.3		6.1 (1)		.02		7.1		.001 (1)		.98	
	Sensory disability	8.5		10.0		7.2 (1)		.01		9.1		4.2 (1)		.04	
	3 or more chronic conditions	14.0		17.2		21.1 (1)		.009		15.0		6.6 (1)		.01	
**SRH^e^**			N/A		N/A		26.6 (5)		<.001		N/A		13.5 (5)		.02
	Excellent	7.0		5.3		N/A		N/A		7.1		N/A		N/A	
	Very good	32.1		29.1		N/A		N/A		32.3		N/A		N/A	
	Good	40		43.2		N/A		N/A		39.8		N/A		N/A	
	Fair	16.9		17.9		N/A		N/A		17		N/A		N/A	
	Poor	3.8		4.3		N/A		N/A		3.7		N/A		N/A	
	Prefer not to say	0.2		0.2		N/A		N/A		0.1		N/A		N/A	
**SRMH^f^**			N/A		N/A		9.7 (5)		.09		N/A		6.5 (5)		.26
	Excellent	26.4		24.7		N/A		N/A		25.7		N/A		N/A	
	Very good	37.0		37.0		N/A		N/A		37.4		N/A		N/A	
	Good	26.3		27.9		N/A		N/A		26.4		N/A		N/A	
	Fair	8.6		9.0		N/A		N/A		8.7		N/A		N/A	
	Poor	1.5		1.5		N/A		N/A		1.7		N/A		N/A	
	Prefer not to say	0.1		0.0		N/A		N/A		0.1		N/A		N/A	

^a^Percentages are weighted and have been rounded and may not total 100.

^b^Older adults who have used of at least 1 type of virtual visit modality in the past 12 months.

^c^Older adults who have expressed the demand for at least 1 type of virtual visit modality.

^d^N/A: not applicable.

^e^SRH: self-rated health status.

^f^SRMH: self-rated mental health status.

**Table 3:** Demographic characteristics of older adults and bivariate associations with the demand for and use of virtual visits, 2011 Canadian Digital Health Survey (N=2303).

Factors		All n (unweighted, weighted)=(2303, 2454)	Use^a^ n (unweighted, weighted)=(1149, 1245)	Versus have not used	Demand^b^ n (unweighted, weighted)=(1773, 1902)	Versus have no interest
**Age (years)**						
	*F* (*df*)	N/A^c^	N/A	1.72 (1)	N/A	3.19 (1)
	*P* value	N/A	N/A	.19	N/A	.07
	Mean (SD)	71.51 (5.13)	71.39 (5.05)	N/A	71.41 (5.04)	N/A
**Gender identity, *χ*^2^ (*df*), *P* value**		N/A	N/A	10.8 (2), .06	N/A	4.6 (2), .46
	Man, weighted %^d^	45.3	42.5	N/A	45.4	N/A
	Woman, weighted %	54.5	57.3	N/A	54.4	N/A
	Other/prefer not to answer, weighted %	0.1	0.3	N/A	0.3	N/A
**Ethnic identity, *χ*^2^ (*df*), *P* value**		N/A	N/A	14.9 (2), .31	N/A	17.9 (2), .16
	White, weighted %	92.1	93.3	N/A	92.9	N/A
	Non-White, weighted %	6.8	4.7	N/A	5.1	N/A
	Other, weighted %	1.5	1.5	N/A	1.2	N/A
	Prefer not to answer, weighted %	1.1	0.6	N/A	1.0	N/A

^a^Older adults who have used of at least 1 type of virtual visit modality in the past 12 months.

^b^Older adults who have expressed the demand for at least 1 type of virtual visit modality.

^c^N/A: not applicable.

^d^Percentages are weighted and have been rounded and may not total 100.

**Table 4:** eHEALS^a^ used to measure eHealth literacy of older adults and bivariate associations with the demand for and use of virtual visits, 2011 Canadian Digital Health Survey (N=2303).

Factor	All n (unweighted, weighted)=(2303, 2454)	Use^b^ n (unweighted, weighted)=(1149, 1245)	Versus have not used	Demand^c^ n (unweighted, weighted)=(1773, 1902)	Versus have no interest
*F* (*df*)	N/A^d^	N/A	2.725 (1)	N/A	13.78 (1)
*P* value	N/A	N/A	.10	N/A	<.001
Mean (SD)	25.95 (6.98)	26.69 (6.81)	N/A	26.59 (6.67)	N/A

^a^eHEALS: eHealth Literacy Scale.

^b^Older adults who have used of at least 1 type of virtual visit modality in the past 12 months.

^c^Older adults who have expressed the demand for at least 1 type of virtual visit modality.

^d^N/A: not applicable.

#### Health-Related Factors

Diagnosed chronic health conditions were assessed with the question “Do you have _____ diagnosed by a health professional?” Respondents who indicated “yes” were counted for each chronic condition. Self-rated mental health status (SRMH) and self-rated health status (SRH) were measured by asking respondents, “In general, how would you rate your overall physical/mental health?” Access to a family doctor was assessed through the question “Do you have a family doctor or regular place of care, such as a health center or a family medical/medicine group?” The responses were dichotomized into yes and no/don’t know.

#### Demographic Factors

Age was calculated based on the respondents’ year of birth and survey date. Gender was self-reported and categorized into man, woman, and other/prefer not to answer. Ethnicity was based on the question “Which ancestry category best describes you?” and responses were collapsed into White, non-White, other, and prefer not to answer.

#### eHealth Literacy

eHealth literacy was measured using the eHEALS, an 8-item self-assessment tool designed to measure respondents’ knowledge, comfort, and perceived skills at finding, evaluating, and applying electronic health information to health problems [11]. Originally developed to assess eHealth literacy levels among youth and youth workers by Skinner and Norman [11], the scale has since then been adapted to a variety of settings, population groups, and multiple languages [12]. Each question measures an aspect of perceived eHealth literacy and is scored on a Likert scale ranging from 1 to 5. Scores are summed to derive an overall eHealth literacy score that ranges from 8 to 40 for each respondent. A higher eHEALS score represents higher self-perceived eHealth literacy.

### Statistical Analysis

#### Bivariate Associations

SPSS Statistics version 24 (IBM) was used for descriptive analyses [13]. Descriptive statistics were calculated for older adults, older adults who used at least 1 type of virtual visit modality in the past year, and older adults who expressed a demand for using at least 1 type of virtual visit modality. Bivariate associations between the demand for and use of virtual visits and socioeconomic, demographic, and health characteristics of older adults were assessed using *χ*^2^ tests for categorical variables and the *t* test for continuous variables. Respondents who used at least 1 modality of virtual visit were compared to respondents who had not used any virtual visit in the past 12 months, and respondents who expressed a demand for at least 1 type of virtual visit were compared to respondents who did not express a demand for any virtual visit modalities.

#### Adjusted Logistic Regressions

SAS version 9.4 (SAS Institute) was used for logistic regression analyses [14]. Using the use and demand for telephone visits, video visits, and secure messaging as outcome variables, multivariable logistic regressions were conducted to assess associations with socioeconomic characteristics of older adults. Socioeconomic characteristics included in the model were household income (below and equal to CAD $80,000 before tax vs above CAD $80,000; note that a currency exchange rate of CAD $1=US $0.78 is applicable), education (less than undergraduate degree vs at least an undergraduate degree), community size (rural vs other), immigration status (not born in Canada vs born in Canada), language at home (English vs other), employment status (retired vs other), and health insurance coverage (no private insurance vs has private insurance). We adjusted for demographic and health factors using gender (male vs female/other), SRH (excellent/very good vs good/fair/poor/prefer not to say), SRMH (excellent/very good vs good/fair/poor/prefer not to say), ethnicity (non-White vs White), and chronic conditions (3 or more vs less than 3). In addition, we included eHealth literacy measured with eHEALS [11] to assess its impacts on the demand for and use of virtual visits. Adjusted models containing all the variables together were used to evaluate the odds of expressing the demand for and use of telephone, secure messaging, or video visits as a function of socioeconomic variables. No interactions were found between gender, SRH, SRMH, income, education, ethnicity, community, immigration status, language, employment status, insurance coverage, number of chronic conditions, and eHealth literacy.

## Results

### Sample Description

All reported percentages and related absolute numbers were weighted. A total of 2303 older adults were surveyed, which represents 19.11% of the total sample of 12,052 from the 2021 Canadian Digital Health Survey. The proportion of older adults who expressed a demand for telephone visits was the highest, followed by video visits and then secure messaging. Similarly, the proportion of older adults in our sample who have used telephone visits within the past year was the highest, followed by video visits and secure messaging. Overall, 1902/2454 (77.51%) older adults expressed a demand for at least 1 modality of virtual visit, and 1245/2303 (54.06%) used at least 1 modality of virtual visits in the past 12 months. The mean age of respondents was 71.51 years (SD 5.13), 1111/2454 (45.27%) respondents identified as male, and 2303/2454 (93.85%) respondents reported having a family physician or a regular place of care. In addition, 30.11% (739/2554) older adults reported having arthritis, followed by diabetes and chronic pain, and 344/2454 (14.02%) respondents reported having 3 or more chronic conditions. Just over one-third of respondents rated their health status as either excellent or very good, and over half of the respondents rated their mental health status as excellent or very good. More than half of the older adults surveyed reported their household income before tax to be below CAD $80,000, over half of the respondents reported not having an undergraduate degree, almost all the respondents self-identified as White, and 9.98% (245/2454) reported living in rural communities. Approximately 82.89% (2034/2454) of respondents were born in Canada, 1867/2454 (76.08%) surveyed respondents reported speaking English at home, 2042/2454 (83.21%) respondents were retired, and 896/2454 (36.51%) respondents had private health coverage. The average eHealth literacy score for older adults in our sample was 25.95 (SD 6.97).

### Bivariate Associations for Demand for and Use of Virtual Care

The proportion of older adults who expressed a demand for at least 1 modality of virtual visits was 1902/2454 (77.51%) respondents, and the proportion of older adults who used at least 1 modality of virtual visits last year with their health care provider was 1245/2303 (54.06%) respondents (Table 1). Bivariate associations between the demand for and use of virtual care and socioeconomic, demographic, and health characteristics are shown in Tables 1-3. Significant associations were found between both income (*P*<.001) and education (*P*<.001) with the demand for virtual visits. No other socioeconomic factors were found to be significantly associated with the demand for virtual visits, except for health coverage (*P*<.001). For the use of virtual visits, a significant association was found with education (*P*=.01) but not for income (*P*=.08). Other socioeconomic factors associated with the use of virtual visits included community size (*P*=.03), home language (*P*=.01), and health coverage (*P*<.001). Compared to older adults who did not express a demand for any virtual visit modalities, older adults who expressed a demand were more likely to have a family physician (1807/1902 [95%] vs 497/552 [90%], *P*<.001), have 3 or more chronic conditions (285/1902 [15%] vs 61/552 [11.1%], *P*=.01), and be diagnosed with chronic pain, drug or alcohol dependence, a learning disability, or a sensory disability. In addition, significant associations were found between the demand for virtual visits and SRHS, household income, education, and health care coverage. Compared to older adults who did not use any modality of virtual visits in the past 12 months, older adults who used virtual visits were also more likely to have a family physician (1199/1245 [96.31%] vs 1100/1209 [90.98%], *P*<.001), have 3 or more chronic conditions (212/1245 [17.03%] vs 133/1209 [11%], *P*<.001), and be diagnosed with chronic pain, diabetes, arthritis, cardiovascular disease, obesity, emotional, a psychological or mental health condition, a physical disability, or a sensory disability. eHealth literacy scores were higher among those who expressed a demand for virtual visits (26.59 vs 23.74, *P*<.001) and higher among those who used virtual visits in the past 12 months (26.69 vs 25.18, *P*=.01).

### Adjusted Logistic Regression Model to Assess Determinants of Demand for Virtual Care

Table 5 displays the adjusted logistic regression findings on factors associated with the demand for telephone visits, video visits, and secure messaging. Socioeconomic factors associated with the use of telephone visits, video visits, and secure messaging were tested with 3 multivariable logistic regression models. Older adults with an annual income of less than CAD $80,000 were less likely to express a demand for video visits (odds ratio [OR] 0.56, 95% CI 0.44-0.72, *P*<.001), secure messaging (OR 0.77, 95% CI 0.61-0.98, *P*=.04), and telephone visits (OR 0.74, 95% CI 0.57-0.97, *P*=.03). Similarly, older adults without an undergraduate degree were less likely to express a demand for video visits (OR 0.62, 95% CI 0.50-0.77, *P*<.001), secure messaging (OR 0.71, 95% CI 0.58-0.88, *P*<.001), and telephone visits (OR 0.68, 95% CI 0.54-0.86, *P*<.001). Other factors that were significant included ethnicity, language, insurance coverage, digital health literacy, and gender. Being non-White and an English speaker at home were negatively associated with the demand for video visits and secure messaging but not for telephone visits. Having no private insurance (OR 1.21, 95% CI 1.01-1.47, *P*=.04) and having more chronic conditions (OR 1.52, 95% CI 1.16-1.99, *P*<.001) were positively associated with the demand for telephone visits. Older adults with higher eHealth literacy, reflected by a higher score on the eHEALS, were more likely to express a demand for video visits (OR 1.04, 95% CI 1.03-1.06, *P*<.001), telephone visits (OR 1.04, 95% CI 1.03-1.06, *P*<.001), and secure messaging (OR 1.05, 95% CI 1.04-1.06, *P*<.001).

**Table 5:** Factors associated with the demand for virtual visits, 2011 Canadian Digital Health Survey (N=2303).

Factors	Video, OR^a^ (95% CI)	Messaging, OR (95% CI)	Telephone, OR (95% CI)
Household income (CAD $80,000 and below vs above CAD $80,000^b^)	0.56 (0.44-0.72)	0.77 (0.61-0.98)	0.74 (0.57-0.97)
Education (less than undergraduate degree vs undergraduate degree or more)	0.62 (0.50-0.77)	0.71 (0.58-0.88)	0.68 (0.54-0.86)
Community (rural vs other)	1.04 (0.79-1.37)	1.05 (0.80-1.38)	1.15 (0.85-1.55)
Immigration status (immigrant/not a citizen vs born in Canada)	1.19 (0.95-1.51)	1.14(0.91-1.44)	1.01 (0.79-1.30)
Language (English vs other)	0.78 (0.64-0.96)	0.71 (0.58-0.86)	1.23 (1.00-1.52)
Employment (retired vs other)	0.91 (0.73-1.13)	0.91 (0.73-1.14)	0.94 (0.74-1.20)
Insurance (without private insurance vs with private insurance)	1.03 (0.86-1.22)	1.00 (0.84-1.19)	1.21 (1.01-1.47)
Gender (male vs female/other)	1.11 (0.93-1.32)	1.19 (1.00-1.41)	0.94 (0.78-1.13)
SRH^c^ (excellent/very good vs good/fair/poor/prefer not to say)	1.15 (0.95-1.39)	1.1 (0.91-1.33)	0.97 (0.79-1.19)
SRMH^d^ (excellent/very good vs good/fair/poor/prefer not to say)	0.95 (0.79-1.15)	0.87 (0.72-1.05)	0.97 (0.79-1.20)
Ethnicity (non-White vs White)	0.59 (0.35-1.00)	0.54 (0.32-0.93)	0.61 (0.36-1.03)
Chronic disease (3 or more vs less than 3)	1.02 (0.81-1.30)	1.1 (0.87-1.40)	1.52 (1.16-1.99)
eHEALS^e^ score (8-40)	1.04 (1.03-1.06)	1.05 (1.04-1.06)	1.04 (1.03-1.06)

^a^OR: odds ratio.

^b^A currency exchange rate of CAD $1=US $0.78 is applicable.

^c^SRH: self-rated health status.

^d^SRMH: self-rated mental health status.

^e^eHEALS: eHealth Literacy Scale.

### Adjusted Logistic Regression Model to Assess Determinants of Use of Virtual Care

Table 6 displays the adjusted logistic regression findings on factors associated with the demand for telephone visits, video visits, and secure messaging. Unlike its associations with demand, a lower income was not significantly associated with the use of secure messaging or telephone visits but was negatively associated with video visits (OR 0.64, 95% CI 0.43-0.95, *P*=.03). A lower level of education was found to be negatively associated with use of telephone visits for older adults (OR 0.78, 95% CI 0.63-0.97, *P*=.02). Other socioeconomic factors that increased the odds of using video visits were being an English speaker (OR 1.99, 95% CI 1.30-3.03, *P*<.001) and being born outside of Canada (OR 1.67, 95% CI 1.18-2.36, *P*<.001). Older adults who were born outside of Canada (OR 1.50, 95% CI 1.04-2.17, *P*=.03) and those who resided in rural communities (OR 1.72, 95% CI 1.12-2.65, *P*=.01) had higher odds of using secure messaging during the past year, while older adults who did not have private insurance had lower odds of using secure messaging (OR 0.73, 95% CI 0.54-0.98, *P*=.04). Interestingly, having no private insurance increased the odds of using telephone visits for older adults (OR 1.41, 95% CI 1.19-1.68, *P*<.001). Being an English speaker was positively associated with use of telephone visits (OR 1.27, 95% CI 1.04-1.55, *P*=.02). Other health and demographic factors associated with the use of telephone visits included having more than 3 chronic conditions (OR 1.55, 95% CI 1.22-1.96, *P*<.001) and being non-White (OR 0.54, 95% CI 0.31-0.92, *P*=.02). Similarly with demand, eHealth literacy was positively associated with the use of video visits (OR 1.03, 95% CI 1.01-1.05, *P*=.01), secure messaging (OR 1.04, 95% CI 1.01-1.06, *P*<.001) and telephone visits (OR 1.03, 95% CI 1.01-1.04, *P*<.001).

**Table 6:** Factors associated with the use of virtual visits, 2011 Canadian Digital Health Survey (N=2303).

Factors	Video, OR^a^ (95% CI)	Messaging, OR (95% CI)	Telephone, OR (95% CI)
Household income (CAD $80,000 and below vs above CAD $80,000^b^)	0.64 (0.43-0.95)	0.70 (0.46-1.07)	0.81 (0.64-1.03)
Education (less than undergraduate degree vs undergraduate degree or more)	0.70 (0.48-1.02)	0.82 (0.56-1.22)	0.78 (0.63-0.97)
Community (rural vs other)	0.95 (0.58-1.56)	1.72 (1.12-2.65)	0.96 (0.73-1.26)
Immigration status (immigrant/not a citizen vs born in Canada)	1.67 (1.18-2.36)	1.50 (1.04-2.17)	1.12 (0.89-1.40)
Language (English vs other)	1.99 (1.30-3.03)	1.23 (0.84-1.80)	1.27 (1.04-1.55)
Employment (retired vs other)	0.97 (0.68-1.40)	1.44 (0.95-2.17)	1.07 (0.86-1.33)
Insurance (without private insurance vs with private insurance)	1.08 (0.80-1.45)	0.73 (0.54-0.98)	1.41 (1.19-1.68)
Gender (male vs female/other)	0.81 (0.60-1.09)	1.03 (0.76-1.40)	0.77 (0.65-0.91)
SRH^c^ (excellent/very good vs good/fair/poor/prefer not to say)	0.86 (0.61-1.20)	0.89 (0.63-1.25)	0.68 (0.56-0.82)
SRMH^d^ (excellent/very good vs good/fair/poor/prefer not to say)	1.03 (0.75-1.43)	0.85 (0.61-1.19)	1.13 (0.93-1.37)
Ethnicity (non-White vs White)	0.55 (0.20-1.53)	0.51 (0.17-1.57)	0.54 (0.31-0.92)
Chronic disease (3 or more vs less than 3)	1.73 (1.21-2.49)	1.27(0.86-1.88)	1.55 (1.22-1.96)
eHEALS^e^ score (8-40)	1.03 (1.01-1.05)	1.04 (1.01-1.06)	1.03 (1.01-1.04)

^a^OR: odds ratio.

^b^A currency exchange rate of CAD $1=US $0.78 is applicable.

^c^SRH: self-rated health status.

^d^SRMH: self-rated mental health status.

^e^eHEALS: eHealth Literacy Scale.

## Discussion

### Principal Findings

This study investigated the demand for and use of virtual care by older adults from a cross-sectional web survey of Canadians during the COVID-19 pandemic. Bivariate associations suggest that older adults’ demand for virtual care is partially driven by the need for health services caused by health conditions (ie, multimorbidity) and partially associated with eHealth literacy levels. Patients with more chronic conditions and worse SRHS were more likely to express a demand for virtual visits. eHealth literacy and socioeconomic factors known to be associated with eHealth literacy among older adults, such as education and income [15,16], were also significantly associated with the demand for virtual visits. The demand for all types of virtual visit modalities was negatively associated with income and education. Older adults with low household income and less education had lower odds to express a demand for virtual visits. In addition, our results suggested that older adults who were non-White and who were English speakers had lower odds to demand video visits and secure messaging. Compared to video visits and secure messaging, a greater proportion of older adults from our sample expressed a demand for telephone visits. The same trend was observed for the use of telephone visits: a greater proportion of older adults have used telephone visits during the past 12 months. We identified multiple socioeconomic, demographic, and health factors associated with the demand for and use of specific virtual care modalities. Specifically, data showed that older adults with lower education and lower income had lower odds of expressing a demand for virtual visits. Additionally, older adults without private insurance coverage had lower odds of using secure messaging. eHealth literacy was a significant predictor of the demand for and use of all modalities of virtual visit.

The literature looking at patient characters and its association with the demand for virtual care among older adults is scarce. A recent report by Health Canada found that individual socioeconomic status, including income and education, plays a key role in influencing access to virtual care [16]. One recent US study looking at interest in telehealth visits for individuals aged 50-80 years found that when compared to individuals with a high school degree or less, those with at least an undergraduate degree are less likely to show interest in telehealth [17]. The same study also found that White individuals have the lowest level of interest [17]. Variations in payment models between Canada and the U.S. could, in part, explain the difference in findings on ethnicity and education. Non-White and individuals with less education in the U.S. could face greater barriers when it comes to paying for in-person visits and other costs associated with an in-person visit (eg, transportation, job flexibility). The Canadian public health care system eliminates direct costs associated with in-person visits, making virtual care a complementary service rather than a substitute to in-person visits. Another US study that investigated the disparities in virtual care use by older adults suggested that non-White patients are less likely to have video visits when compared to White patients [18]. The association between ethnicity and the use of virtual visits seems to be more nuanced than what has been studied thus far. Additional research is needed to ensure that older adults who are ethnic minorities have access to all types of virtual care modalities.

Lower reported usage of video visits and secure messaging services may be related to financial and technological barriers, such as a lack of digital equipment, internet access, and al ack of skills to navigate technology [19]. Consistent with the past literature, participants with low household income are less likely to conduct a video visit [20,21]. A recent Infoway analysis demonstrated that higher-income groups were more likely to use virtual care when compared to lower-income groups [22]. Considering that some video visits were offered by private and for-profit vendors during the pandemic, it is likely that income would become a barrier to using virtual health technologies that are not covered under the public payment plan. In addition, cohort research looking at virtual care usage during the pandemic has suggested a digital divide between telephone and video use based on race, income, and age. Studies have found that older, lower-income individuals use more telephone, while White, higher-income individuals use more video [18,20,23]. The proliferation of private services could also explain the association between insurance status and the use of secure messaging. In response to COVID-19, temporary billing codes were established by all provinces and territories, with the exception of Nunavut [24]. Most provinces provide billing codes to cover synchronous visits through telephone and video, but coverage for secure messaging is sparse [24]. The use of secure messaging services might therefore be limited by private health insurance coverage, as our study suggests. This could also explain the positive association between chronic conditions and the use of video and telephone visits. Older adults with multiple chronic conditions would likely require more health services, and virtual visits as well. The financial barriers associated with the use of secure messaging might explain the lack of significant associations between secure messaging and chronic condition status.

In line with past research, our data show that eHealth literacy is an important driver/constraint for the use of virtual care [15,25]. The past literature on the digital divide suggests that older adults disproportionally suffer adverse consequences from a lack of technological access and literacy [15,26]. Older adults typically face challenges in accessing virtual care due to a lower use of digital health technologies, a lack of motivation to use technology, and a lack of technological equipment and broadband access [20,25]. This could explain the low prevalence for the use of secure messaging and video visits from our study. Secure messaging and video visits are more complex technologies when compared to telephone visits and require the users to have higher levels of digital as well as digital health literacy, which might pose as barriers of access for older adults [27]. In addition, physical barriers, such impaired cognition, hearing, vision, and dexterity, may also cause problems for older adults in using more complex technologies, such as video visits and secure messaging [19]. The observed prevalence is consistent with findings from the U.S., suggesting that older age was associated with lower usage of video and telephone usage during the pandemic [20] and that the majority of virtual visits conducted by older adults during the COVID-19 pandemic were via audio technologies [18]. Older adults from marginalized groups may face additional challenges using complex technologies due to language barriers and income constraints. Patient outcome studies have suggested that the use of telemedicine among older adults can lead to high levels of patient satisfaction and acceptance [19]. Unless programs and policies are put in place to promote digital health technology uptake among older adults, exacerbating the current digital divide will likely lead to more inequities. Our finding adds to the emerging evidence base advocating for improved patient eHealth literacy to close the digital divide and the associated inequalities.

### Limitations

Our study suffers from a few limitations. The Canadian Digital Health Survey is a web survey and therefore may limit participation by older adults with limited access to technological equipment and the internet. Therefore, our findings might skew toward older adults with more internet and technology access. Second, the study population was weighted to render a representative sample of the Canadian population. As a result, our sample was predominantly White and therefore may have had more access to technology than other ethnic/culture groups. We did not collect information on the duration or completion of the health encounter and therefore cannot assume that these virtual visits were all successfully completed. The chronic condition of respondents was self-reported. Although our survey question prompted respondents to only report chronic conditions as diagnosed by a health care professional lasting for more than 6 months, it is possible that respondents reported self-diagnosed chronic conditions. Health care utilization was self-reported and may be impacted by recall error, although past research has shown that bias and variance of recall error of health care usage were minimized for the 12-month recall period [28].

### Conclusion

Despite limitations, this study provides novel insights into potential drivers and barriers that determine the demand for and use of virtual visits among older adults during the COVID-19 pandemic. We found that despite high levels of demand to access virtual visits among older adults, the rates of usage are much lower, especially for video visits and secure messaging. Lower usage of complex technologies could be caused by financial barriers, inadequate eHealth literacy, a lack of technological equipment and broadband access, and physical limitations. In addition, socioeconomic inequities associated with the use of secure messaging and video services emphasize the need to regulate the proliferation of private, for-profit virtual care vendors. Other socioeconomic and demographic disparities, such as ethnicity, immigration status, and education, that may pose challenges to accessing virtual visits for older adults should be carefully investigated to reduce existing inequities in health service access and health outcomes. Future studies should test the extent to which virtual care can deliver improvements in access to health care services as well as patient experience among older adults.

## Abbreviations

eHEALS: eHealth Literacy Scale
Infoway: Canada Health Infoway
LEO: Léger Opinion
OR: odds ratio
SRH: self-rated health status
SRMH: self-rated mental health status
